# Supplementary material for: Automatic Recognition of Ragged Red Fibers in Muscle Biopsy from Patients with Mitochondrial Disorders
Source: Healthcare (Basel). 2022 Mar 19;10(3):574. doi: 10.3390/healthcare10030574 (PMC8949467; doi:10.3390/healthcare10030574)
Supplement: Supplementary file 1 [file healthcare-10-00574-s001.zip › healthcare-1591106-supplementary.pdf]

Supplementary Materials:

Table S1. Clinical, histological, and molecular features of the nine patients.

	**Patient 1**	**Patient 2**	**Patient 3**	**Patient 4**	**Patient 5**	**Patient 6**	**Patient 7**	**Patient 8**	**Patient 9**
Sex	M	M	F	F	M	M	F	M	F
Age at muscle biopsy	5 yrs	11 yrs	41 yrs	8 yrs	60 yrs	3 months	46 yrs	67 yrs	58 yrs
Clinical features	Cardiomyopathy, myopathy	Retinitis pigmentosa, ptosis, ataxia, hearing loss	Cardiomyopathy, hearing loss, diabetes	Retinitis pigmentosa, hearing loss, diabetes, psychomotor regression	Ophthalmoplegia	Hypotonia, severe metabolic acidosis	Ptosis, decreased visual acuity	Ophthalmoplegia	Ophthalmoplegia
Histology	RRFs; subsarcolemmal rims, lipid storage	RRFs, COX-negative SDH-positive fibers, lipid storage	RRFs, COX-negative SDH-positive fibers	RRFs, COX-negative SDH-positive fibers, lipid storage	Variation in fiber size, RRFs, COX-negative SDH-positive fibers	RRFs, diffuse reduced COX staining, SDH-positive fibers	RRFs, COX-negative SDH-positive fibers	RRFs, COX-negative SDH-positive fibers	RRFs, COX-negative SDH-positive fibers, lipid storage
Number of analysed images	2	4	6	5	5	2	2	6	5
Mutation	ACAD9 c.170C>T (p.Pro57Leu)+ c.1240C>T (p.Arg414Cys)	8 Kb mtDNA single deletion	m.5522G>A MT-TW (tRNATrp)	7 Kb mtDNA single deletion	mtDNA common deletion	Muscular mtDNA depletion	OPA1 c.124C>T (p.H42Y)	mtDNA common deletion	5 Kb mtDNA single deletion
Diagnosis	Mitochondrial Cardiomyopathy	Kearns-Sayre syndrome	Encephalocardiomyopathy	Kearns-Sayre syndrome	CPEO	Mitochondrial DNA depletion syndrome	DOA, dominant optic atrophy plus syndrome	CPEO	CPEO

Abbreviation: M, man; F, woman; CPEO, chronic progressive external ophthalmoplegia; RRFs, ragged red fibers; COX, cytochrome c oxidase.; SDH, succinate dehydrogenase, mtDNA, mitochondrial DNA.

Table S2. Hyperparameters values for each model after the tuning operation.

	**Waste-tissue classification**	**Ragged – not ragged classification**
**RF**	**min size of nodes**: 15<br>**number of trees**: 625<br>**number of variables:** 9<br>**priors of the classes:** 0.487, 0.316	**min size of nodes**: 19<br>**number of trees**: 1466<br>**number of variables:** 4<br>**priors of the classes:** 0.322, 0.792
**GBM**	**distribution:** adaboost	**distribution:** adaboost

	**number of trees:** 300<br>**shrinkage:** 0.483373<br>**fraction selected:** 0.630<br>**fraction for train:** 0.946	**number of trees:** 173<br>**shrinkage:** 0.0540611<br>**fraction selected:** 0.514<br>**fraction for train:** 0.988
**SVM**	**kernel:** radial<br>**degree:** -<br>**gamma:** 0.01572429<br>**coef0:** -<br>**cost:** 5.31966	**kernel:** radial<br>**degree:** -<br>**gamma:** 0.01732987<br>**coef0:** -<br>**cost:** 4.905418

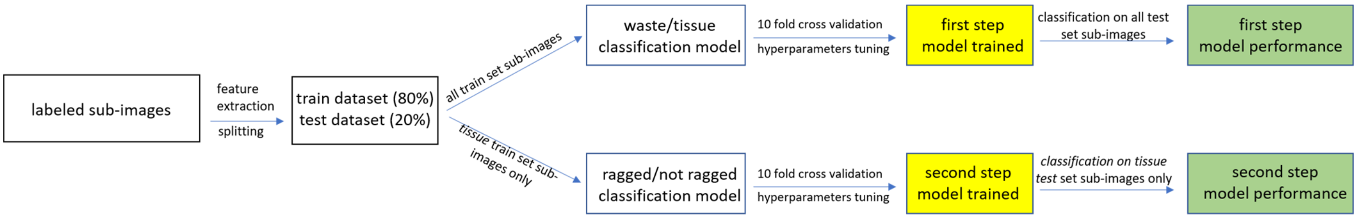

Figure S1: protocol used to select the model with the best overall fit for both classification steps.
